# Brain Diseases

**Published:** 1885-05

**Authors:** 


### Brain Diseases.

Dr. Reuben A. Vance, Physician-in-Chief of the New York Institute for the Paralyzed and Epileptic, also Attending Physician for Diseases of the Nervous System, at the Out-Door Department of Bellevue Hospital, New York, says: "In the treatment of these early symptoms of cerebral disease, some preparation of phosphorus is imperatively demanded. The preparation on which I place the most reliance is Professor Horsford's Acid Phoshate, either singly or combined with a proper amount of quinine. This latter drug I use with gratifying effect in cases where the patients are distressed by slight variations of temperature, and in which the peripheral circulation is weak and languid. In all other cases the Acid Phosphate is prescribed pure, and the results obtained are gratifying in every respect. The Acid Phosphate not only does not create any difficulty with digestion, but is of the greatest service where such difficulties exist. No trouble need be anticipated from its prolonged exeibition, as its excess is immediately carried off by the kidneys."

